# Long-term effect of respiratory training for chronic obstructive pulmonary disease patients at an outpatient clinic: a randomised controlled trial

**DOI:** 10.1186/s40169-015-0073-2

**Published:** 2015-10-12

**Authors:** Fang Xi, Zheng Wang, Yong Qi, Richard Brightwell, Peter Roberts, Angus Stewart, Moira Sim, Wei Wang

**Affiliations:** The People’s Hospital of Zhengzhou University, 450003 Zhengzhou, China; School of Medical Sciences, Edith Cowan University, Perth, WA 6027 Australia; Beijing Municipal Key Laboratory of Clinical Epidemiology, School of Public Health, Capital Medical University of Medical Science, 100069 Beijing, China

## Abstract

**Objective:**

To assess the effect of respiratory training (RT) on lung function, activity tolerance and acute exacerbation frequency with chronic obstructive pulmonary disease (COPD).

**Design:**

A randomised controlled trial.

**Setting:**

Outpatient clinic and home of the COPD patients, Zhengzhou City, China.

**Subjects:**

Sixty participants with COPD were randomised into two groups: an intervention group (*n* = 30) which received the RT in self-management and a control group (*n* = 30) that received an education program during the study.

**Intervention:**

Pulmonary function, activity tolerance and frequency of acute exacerbation of these COPD patients were evaluated before and after the program. The intervention and control programs were delivered at monthly outpatient clinic visits over a period of 12 months. The pulmonary rehabilitation (PR) program was conducted by a physiotherapist (who delivered RT to the participant over a minimum of 1 h per visit) for the intervention group, whereas the control group received routine health education provided by physiotherapists. The intervention group patients were then instructed to perform exercises at home as taught in the RT at least 5 days per week at home.

**Results:**

After 12 months of RT, the lung function and the activity tolerance of the COPD patients in the intervention group were significantly improved and the exacerbation frequency was also decreased.

**Conclusion:**

Long-term RT can improve lung function and activity tolerance while decreasing the frequency of acute exacerbation for COPD patients.

## Background

Chronic obstructive pulmonary disease (COPD) is a lung disease characterised by chronic obstruction of lung airflow which interferes with normal breathing and is not fully reversible [1]. It is associated with a significant reduction in physical activity and psychological problems, which contribute to disability and poor health-related quality of life (HRQoL) [2]. COPD is a common cause of illness and death that affects a large and increasing number of individuals in both developed and developing countries. Estimates suggest that COPD will become the 3rd most common cause of death in the world and 5th most common cause of illness in the next 20 years [3, 4]. Unfortunately, to-date there is no effective therapy which can reverse the decline in lung function in COPD and the only intervention which can reduce the rate of decline is smoking cessation. All existing evidence-based clinical practice guidelines recommend the use of pulmonary rehabilitation for the management of COPD [5, 6]. PR is an evidence-based, multidisciplinary and comprehensive intervention for symptomatic patients who may have decreased functional status [7]. Numerous high-quality trials have shown that PR, consisting of exercise training, self-management education, nutritional care and psychosocial management, improves exercise capacity, symptoms and health-related quality of life and reduces health-care costs [4, 8, 9]. Despite this, many people with COPD do not continue their PR program, and long-term maintenance has been difficult to achieve after short-term treatment [10]. Although PR therapy including supervised exercise training, nutritional support, self-management education, and psychosocial support is effective for COPD patients, few studies have focused on follow up beyond 12 months and long term outpatient compliance in a RT program. The effect of RT for COPD patients is positive. For example, COPD-X guildlines [11] and Lin et al. [12] reported the effectiveness of RT that PR reduces COPD hospitalization. Moreover, few PR studies focused on acute exacerbation frequency with COPD [13, 14].

This present study aimed to evaluate the impact of a 12-month RT program on lung function, activity tolerance and frequency of acute exacerbations. Pulmonary function, activity tolerance and frequency of acute exacerbation of these COPD patients were evaluated before and after the program during Feb 2010 to April 2012.

## Methods

### Study design

All participants confirmed their informed consent in writing, and this study was approved by the Ethical Committee of Henan Province People’s Hospital. The study was performed in accordance with the ethical standards laid down in the 1964 Declaration of Helsinki.

**Fig. 1:**
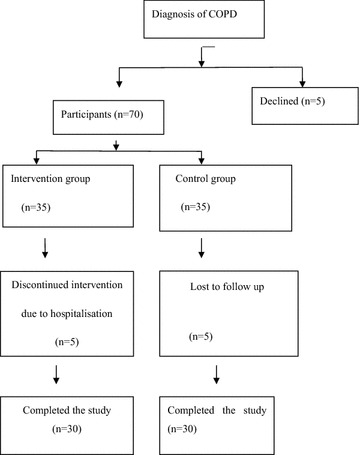
Flowchart of participants

The patients were screened in the Respiratory Department of Henan Province People’s Hospital, China. The patients were randomised by computer and allocated to two groups: intervention and control. Participants visited the clinic monthly for the PR and education during this 12-month program. Pulmonary function, activity tolerance and frequency of acute exacerbation of these COPD patients were evaluated before and after the program (Fig. 1).

The sample size of this study was estimated by calculation based on the data collected from preliminary experiments. The minimum sample size of 23 in each group achieves 91 % power to detect a difference of −5.0 between the null hypothesis that both group means are 5.0 and the alternative hypothesis that the mean of group 2 is 10.0 with estimated group standard deviations of 5.0 and 5.0, and with a significance level (alpha) of 0.05000 using a two-sided two-sample *t* test.

### Patients

The inclusion criteria:

- COPD was diagnosed based on the GOLD classification [3];
- COPD clinically stable 8 weeks prior to the study (i.e., no symptoms of increased phlegm production or phlegm purulence).

The exclusion criteria:

- hospitalisation;
- presence of neuromuscular disease;
- associated respiratory disease;
- orthopaedic or neurological disease that affected gait;
- In the last 3 months impairment due to co-morbidities, such as myocardial infarction, heart failure, stroke or neoplasm;
- prior pneumonectomy or other thoracic surgery.

The presence of stable co-morbidities was not considered an exclusion criterion as most patients with COPD are elderly and commonly affected by other multiple co-morbidities [15].

### Lung function measurements

Lung function parameters were collected pre and post RT with MIR Spirobank lung function spectrometry (Spirobank, Italy). Forced vital capacity (FVC), the forced expiratory volume in 1 s (FEV1) and FEV1/FVC were evaluated. Exacerbation frequencies were documented during the 12-month study. Acute exacerbation was defined by exacerbation of cough, dyspnoea or wheezing and increased production of sputum, with or without fever. Two periods of exacerbation in 1 week were documented as one exacerbation. The physiotherapist followed up the participants’ status by a telephone call once a week during the first 2 months and then once every 2 weeks subsequently. During the phone call, the physiotherapist guided the PR (see more details in the following section of “Educational and RT Programs”) and informed the relatives about the importance of surveillance. Patients re-visited the hospital every month. Patients were individually guided, monitored and the PR was increased closely in line with the health status.

### Interventions

#### Educational program

In accordance with the GOLD guidelines, patients in both groups were treated with inhaled bronchodilators and corticosteroids, and nutrition support therapy as required [3]. The control group in their once monthly visit to the outpatient clinic received routine health education from a physiotherapist about the development and progression of COPD. Compact discs and brochures were distributed to the patients and their relatives, in the hope that they could learn and continue the PR. The patients were required to revisit the outpatient clinic monthly where they were interviewed and recorded while they also got a general medical review at their clinic appointments.

#### Respiratory training (RT) program

The intervention group received the RT program. The RT method was based on studies by Collins et al. [16], Tiep [17] and Mendes de Oliveira et al. [18]. Briefly, the method was performed by a combination of pursed-lip breathing, abdominal breathing (diaphragmatic breathing) and upper and lower limb exercises. The respiratory training included pursed-lip breathing using the following methods: inhaling through the nose while silently counting from one to two, and then pursing the lips to slowly exhale while silently counting from 1–4, 5 min at a time, three times per day.

The abdominal breathing (diaphragmatic breathing) exercise included inflating the abdomen while inhaling and deflating the belly while exhaling. While practising, the participants placed one hand on the chest and the other on the abdomen. Then they relaxed and bloated the abdomen when inspiring; tightened up and contracted the abdomen when expiring. This was performed for 10 min each time and twice per day in the morning and evening, and was increased in terms of the lasting time of the exercise until it became the normal method for breathing throughout the day.

The upper limb exercises were performed with hand weights while seated comfortably and consisted of ten repetitions of elbow flexion, ten repetitions of elbow abduction, ten repetitions of shoulder abduction and ten repetitions of shoulder flexion. Upper limb training was performed daily beginning with a duration of 5 min each time, gradually increasing to 20 min. The lower limb exercises were performed with ankle weights while patients were seated comfortably and consisted of ten repetitions of hip flexion and ten repetitions of knee extension, initially for 10 min per day and gradually increasing up to 20 min each day.

A minimum of 1 h of the exercise was performed on their monthly visit at the outpatient clinic and participants were advised to continue their exercises at home for a minimum of 5 days per week. The participants were required to attend the outpatient clinic monthly where they underwent clinical assessment and interviews as outlined below.

### Physical measurements

At monthly outpatient clinic visits, clinical measurements, tests and surveys were performed blind by assessors. Participants’ height and weight were measured. The modified Medical Research Council (MMRC) dyspnoea scale [3], COPD assessment test (CAT) and the Saint George’s respiratory questionnaire (SGRQ) were applied [19]. The 6-Minute Walk Test (6MWT) was conducted in a 50 metre long corridor. During 6MWT the patients were instructed to walk as fast as possible for 6 min and to then decrease speed or interrupt the test if experiencing severe dyspnoea or any other discomfort. Heart rate, dyspnoea, and oxygen saturation were measured before and after each test. The participant was guided and encouraged to complete the test. The body mass, airflow obstruction, dyspnoea and exercise capacity index (BODE index) for COPD was calculated accordingly [20]. A COPD assessment test (CAT) was performed by an experienced observer. The statistician who performed the data analysis blind from all the clinical tests.

### Statistical analysis

SPSS 15.0 software (SPSS Inc, Chicago, IL) was used for statistical analysis. Data were reported as mean ± SD for continuous variables and frequencies for categorical variables. *P* values of <0.05 were considered statistically significant.

## Results

### Baseline characteristics

Seventy-five COPD patients were eligible for enrolment, out of which 70 consented and were randomised into the RT and control groups respectively. After group assignment, ten patients (five from each group) were hospitalised because of respiratory infections during the study and had to be excluded. Following informed consent, 60 COPD patients were randomised and divided into two groups, an intervention group (*n* = 30) which received the RT and a control group (*n* = 30) which received an education program during the study. In the end, the study was completed with groups of participant size 30 each.

As shown in Table 1, the mean FEV1 was 41.9 % (±2.6 %) in the intervention group versus 43.33 % (±3.6 %) in the control group; the mean FVC was 68.7 % (±15.5 %) in the intervention group versus 65.3 % (±14.8) in the control group. The age, sex, education background, disease course and lung function parameters were not significantly statistically different between the intervention and control groups (*P* > 0.05).

**Table 1:** Demographic characteristics of the patient population

Characteristics	Control group (n = 30)	Intervention group (n = 30)	χ^2^ test* t test	*P* value
Age, year	73.0 ± 5.8	75.4 ± 6.6	0.60*	0.439
Female	7	8	0.09	0.764
Ex-smokers	22	24	0.37	0.543
Smoking index (pack year)	26.2 ± 4.6	25.3 ± 4.8	0.44*	0.562
Hypertension	14	12	0.27	0.603
Coronary artery disease	7	6	0.10	0.751
Stroke	4	4	0.14	0.708
Type 2 diabetes	3	4	0.001	0.974
Numbers of concomitant diseases	2.3 ± 0.7	2.2 ± 1.0	0.53*	0.467
Resting blood gas findings (FiO_2_ 21 %)				
PaO_2_ <60 mmHg	3	2	0.001	0.467
PaCO_2_ >45 mmHg	4	6	0.48	0.488
PH <7.35	2	1	0.001	0.974
FVC (% of predicted)	65.3 ± 14.8	68.7 ± 15.5	1.05*	0.306
FEV1 (% of predicted)	43.33 ± 3.6	41.9 ± 2.6	0.616*	0.533
50–80	3	5	0.14	0.708
30–50	11	10	0.07	0.791
~30	16	15	0.07	0.791

*FVC* forced vital capacity, *FEV1* forced expiratory volume in one-second

### The effect of PR on symptoms

Table 2 reflects the changes in CAT score, SGRQ score, 6MWT distance, BODE index and MMRC score. A decrease of two points (from six to four) in the score for the BODE index for the patients in the intervention group was noted. This change was due to the decrease in dyspnoea in the intervention group, whereas we did not find such a BODE index decrease in the control group. The BODE index and the 6MWT distance were significantly different between the intervention group and the control group after pulmonary rehabilitation (*P* < 0.05 in each situation).

**Table 2:** The effect of PR on symptoms

	Control group (n = 30)		Intervention group (n = 30)	
	Before rehabilitation	After rehabilitation	Before rehabilitation	After rehabilitation
CAT score	14 ± 2.4	8 ± 2*	14.6 ± 2.5	7.2 ± 1.8*
SGRQ score	42.33 ± 4.6	63.66 ± 3.6*	38.2 ± 3.6^#^	59.6 ± 5.4*
6MWT distance (m)	273 ± 19	281 ± 22	284 ± 18	330 ± 19*^,&^
BODE index	6 ± 0.44	4 ± 0.38*	6 ± 0.7	6 ± 0.5^&^
MMRC score	2 ± 0.6	1 ± 0.8*	2 ± 0.7	1 ± 0.5*

* *P* < 0.05 compared with that before rehabilitation. Data are presented as mean ± SD

^#^ *P* < 0.05 compared with that of the control group before rehabilitation

^&^ *P* < 0.05 compared with that of the control group after rehabilitation

### The effect of PR on lung function

The lung function parameters before and after interventions are shown in Table 3. There was no significant change in any of the pulmonary function parameters pre and post in the control group. However, RT improved FEV1, FVC and FEV1/FVC in the intervention group (*P* < 0.05 in each situation). FEV1 was significantly different between the intervention group and the control group after pulmonary rehabilitation (*P* < 0.05).

**Table 3:** The effect of the rehabilitation program on lung function

	Control group (n = 30)		Intervention group (n = 30)	
	Before rehabilitation	After rehabilitation	Before rehabilitation	After rehabilitation
FEV1(L)	1.45 ± 0.17	1.43 ± 0.19	1.41 ± 0.16	1.52 ± 0.16*^,&^
FVC(L)	2.42 ± 0.19	2.45 ± 0.20	2.42 ± 0.20	2.46 ± 0.19*
FEV1/FVC (%)	60.53 ± 6.68	59.53 ± 6.89	58.03 ± 6.99	60.43 ± 6.88*

* *P* < 0.05 compared with that before rehabilitation. Data are presented as mean ± SD

^&^ *P* < 0.05 compared with that of the control group after rehabilitation

### The effect of PR on acute exacerbation frequencies

The acute exacerbation frequencies were 1.30 (SD ± 1.02) in the intervention group compared with 2.40 (±1.30) times in the control group, which was statistically significantly different between both groups (t = −3.639, *P* < 0.01).

## Discussion

PR is an evidence-based, multidisciplinary program whose main components are supervised exercise training, nutritional support, self-management education, and psychosocial support. It could increase the patients’ endurance limit due to hypoxia, relieve the symptoms, improve their exercise capabilities and quality of life, and has become a major part of therapy in stabilising COPD. However, the availability of PR programs in most countries is limited and few patients are enrolled into such a treatment modality [21–23]. Moreover, PR carried out in a clinical setting and based on hospital guidelines requires qualified health care professionals, equipment and facilities, which are expensive to maintain and this cost contributes toward the reduction in the availability of PR at health care providers [24]. COPD patients are oftern reduce their activities due to progressive, irreversible airway obstruction which lowers their exercise capacity and quality of life. Some even require home care due to significant dyspnoea. Exercise training is the core of lung function rehabilitation [25], which can improve patients' exercise endurance, alleviate their dyspnoea, improve their ventilation burden and finally, facilitate the rehabilitation of the whole body.

This study randomly assigned COPD patients to intervention and control groups to evaluate the effects of RT. The results showed that RT significantly improves the parameters of pulmonary function, including FVC, FEV1 and FEV1/FVC (%). The results for FVC are similar to the study by Ngaage et al. [26], who showed that patients with COPD have similar outcomes after physical training for the upper and lower extremities and diet education. Pursed-lip respiration could increase the inspiration maneuver, slow down the expiration air flow, and reduce the residual lung volume. Breath-holding for two or 3 s could improve the distribution of air in the alveoli and alleviate patients’ dyspnoea.

Abdominal breathing elevates the diaphragm and, thus, improves lung function, and the results of this study are similar to those reported by Lin et al. [27], who evaluated the effects of respiratory training in 40 patients with COPD, and Chao et al. [28] who evaluated modified abdominal breathing in 25 patients with COPD. These data suggested that RT could improve the strength and endurance of respiratory muscles, and improve lung functions.

The 6MWT is a widely accepted method for determining functional capacity for exercise, being commonly used in field studies due to its low-cost, reproducibility, objectivity and easy application in relation to patient age and education level [5, 29]. The intervention of RT in this study improved the 6-min walking distance. These results are consistent with the results reported from Spencer et al. [30] and Magadle et al. [31].

The frequency of exacerbations is an important outcome indicator for COPD. Acute exacerbation of COPD (AECOPD) can signify ineffective treatment and frequent episodes of AECOPD are associated with increased frequency and duration of hospitalisation, poorer quality of life, and poorer prognosis. This study showed that RT could improve lung function, delay the degeneration of lung function, and improve the efficacy of respiration, thus potentially reducing the frequency of disease exacerbation and treatment cost, as well as improving prognosis.

## Limitations

Our intended sample size was at least 60, with 30 in each group, based on the power calculations and pragmatic decisions on what was achievable. With the loss to follow up we still achieved the required minimum of 23 participants in each group. We acknowledge this small sample size could influence the analysis with limited statistical power (91 %) to detect the difference. In addition, we omitted the use of SaO2/SpO2 values before and after 6MWT, which would have provided useful information about the functional status of the participants.

## Recommendations

Healthcare workers should be well trained in management of COPD, with the ability to assess, diagnose, treat or refer, and educate patients and their caregivers and this is particularly vital for a country like China which is marching rapidly into an ageing society [32, 33]. A key policy priority should therefore be to plan for the long-term care of people with COPD and the delivery of PR in community-based care. Additionally, more research should be done to improve understanding of the different social and environmental risk factors and the ecology of healthcare for chronic disease control and management [34].

## Conclusion

The results of this study showed that long term (12 months) RT can improve lung function and activity tolerance, and decrease frequency of acute exacerbation for COPD patients. RT as a simple to learn, cost-effective method is worthy of promotion.
